# Systematic impacts of chronic unpredictable mild stress on metabolomics in rats

**DOI:** 10.1038/s41598-020-57566-x

**Published:** 2020-01-20

**Authors:** Chunmei Geng, Yujin Guo, Changshui Wang, Dehua Liao, Wenxiu Han, Jing Zhang, Pei Jiang

**Affiliations:** 1 Jining First People’s Hospital, Jining Medical University, Jining, 272000 China; 2 Department of Clinical Translational Medicine, Jining Life Science Center, Jining, 272000 China; 3 Department of Pharmacy, Hunan Cancer Hospital, Central South University, Changsha, 410011 China; 4 Department of Medical Engineering, Jining Medical University, Jining, 272000 China

**Keywords:** Diagnostic markers, Predictive markers, Biomarkers, Neuroscience, Diseases of the nervous system, Depression

## Abstract

Depression is the most common disabling psychiatric disease, with a high prevalence and mortality. Chronic unpredictable mild stress (CUMS) is a well-accepted method used to mimic clinical depression. Recent evidence has consistently suggested that the cumulative effects of CUMS could lead to allostatic overload in the body, thereby inducing systemic disorders; however, there are no previous systematic metabonomics studies on the main stress-targeted tissues associated with depression. A non-targeted gas chromatography–mass spectrometry (GC–MS) approach was used to identify metabolic biomarkers in the main stress-targeted tissues (serum, heart, liver, brain, and kidney) in a CUMS model of depression. Male Sprague–Dawley rats were randomly allocated to the CUMS group (*n* = 8) or a control group (*n* = 8). Multivariate analysis was performed to identify the metabolites that were differentially expressed between the two groups. There were 10, 10, 9, 4, and 7 differentially expressed metabolites in the serum, heart, liver, brain and kidney tissues, respectively, between the control and CUMS groups. These were linked to nine different pathways related to the metabolism of amino acids, lipids, and energy. In summary, we provided a comprehensive understanding of metabolic alterations in the main stress-targeted tissues, helping to understand the potential mechanisms underlying depression.

## Introduction

An increasing body of evidence has revealed that the cumulative effect of stress can trigger allostatic overload^1–3^. “Allostatic load” refers to the effects of prolonged continuous or intermittent activation of effectors involved in allostasis^4–6^. Allostatic load has been associated with many diseases, such as cardiovascular disease, diabetes, stroke, chronic kidney disease, abdominal obesity, and depression^7,8^. These all involve the whole body. Thus, the stress-induced allostatic overload involved in depression is focused on in our study.

Depression is a seriously debilitating psychiatric disease, characterized by high mortality and morbidity. The chronic unpredictable mild stress (CUMS) model, a well-accepted animal model of depression, is used to explore the mechanisms underlying depression^9–11^. One proposed mechanism involves allostatic load, a multidimensional biologic construct that involves biomarkers across the physiologic domains of neuroendocrine, autonomic, immune, and metabolic function^7^. Allostatic overload resulting from CUMS in animal models causes atrophy of neurons in the hippocampus and prefrontal cortex^4,5^, myocardial ischemia^9^, abnormal hepatic metabolism^12^, and poor kidney outcomes^8,13^. Thus, the impact of stress involves the whole body and depression is linked to multiple diseases such as cardiovascular disease^9^. However, there are no previous systematic metabonomics studies focusing on the main stress-targeted tissues.

Thus, the aim of the present study was to provide a panoramic and systematic view of metabolic alterations in stress-targeted tissues (serum, heart, liver, brain, and kidney) in the context of the CUMS-induced rat model of depression. To this end, a gas chromatography–mass spectrometry (GC-MS)-based metabolomics approach coupled with univariate and multivariate analyses was performed to identify metabolic biomarkers in the stress-targeted tissues related to depression in order to develop insights into the metabolic pathogenesis underlying depression.

## Results

### Behavioral tests

**Figure 1:**
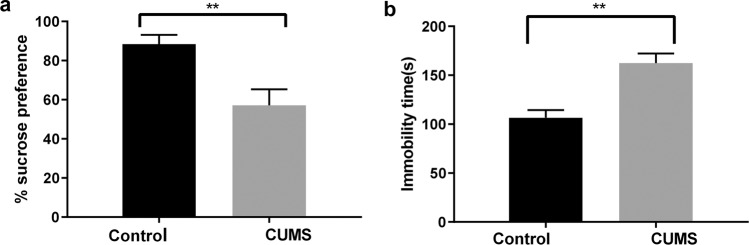
Depression-like behaviors were evaluated by (**a**) the sucrose preference test, and (**b**) the forced swimming test. Data are the means ± SD (*n* = 8). **p < 0.01 CUMS control when compared to the control group.

After four weeks of the CUMS procedures, the CUMS rats showed a significantly lower mean sucrose preference percentage in the SPT (57.12% ± 8.22%) compared to the percentage in the control group (88.34% ± 4.82%; *n* = 8, *p* < 0.0001). In addition, CUMS led to a longer mean immobility time in the FST (162.38 ± 9.85 s) compared to the time in the control group (106.63 ± 7.76 s; *n* = 8, *p* < 0.0001). The results were as shown in Fig. 1, indicating that the model of depression was successfully established.

### GC-MS chromatograms of serum and tissue samples

**Figure 2:**
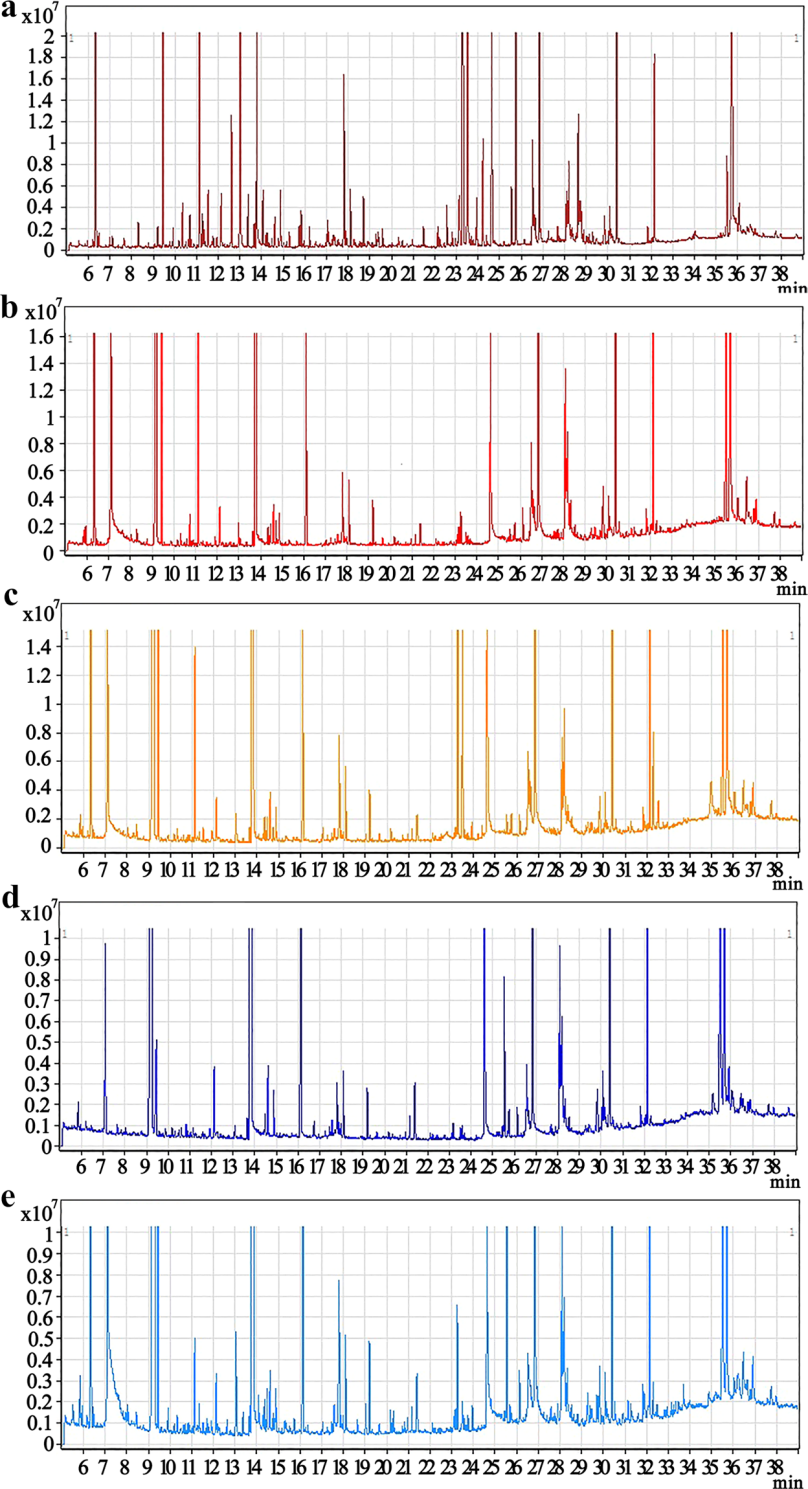
Representative GC–MS total ion current (TIC) chromatograms of the serum (**a**), heart tissue (**b**), liver tissue (**c**), brain tissue (**d**), and kidney tissue (**e**) samples from a mixture of the CUMS and control rats.

Representative GC–MS total ion current (TIC) chromatograms of the quality control (QC) serum and tissue samples (heart, liver, brain, and kidney) from a mixture of the CUMS and control rats all showed strong signals and good RT reproducibility (Fig. 2).

### Multivariate statistics of the metabolomics data

**Figure 3:**
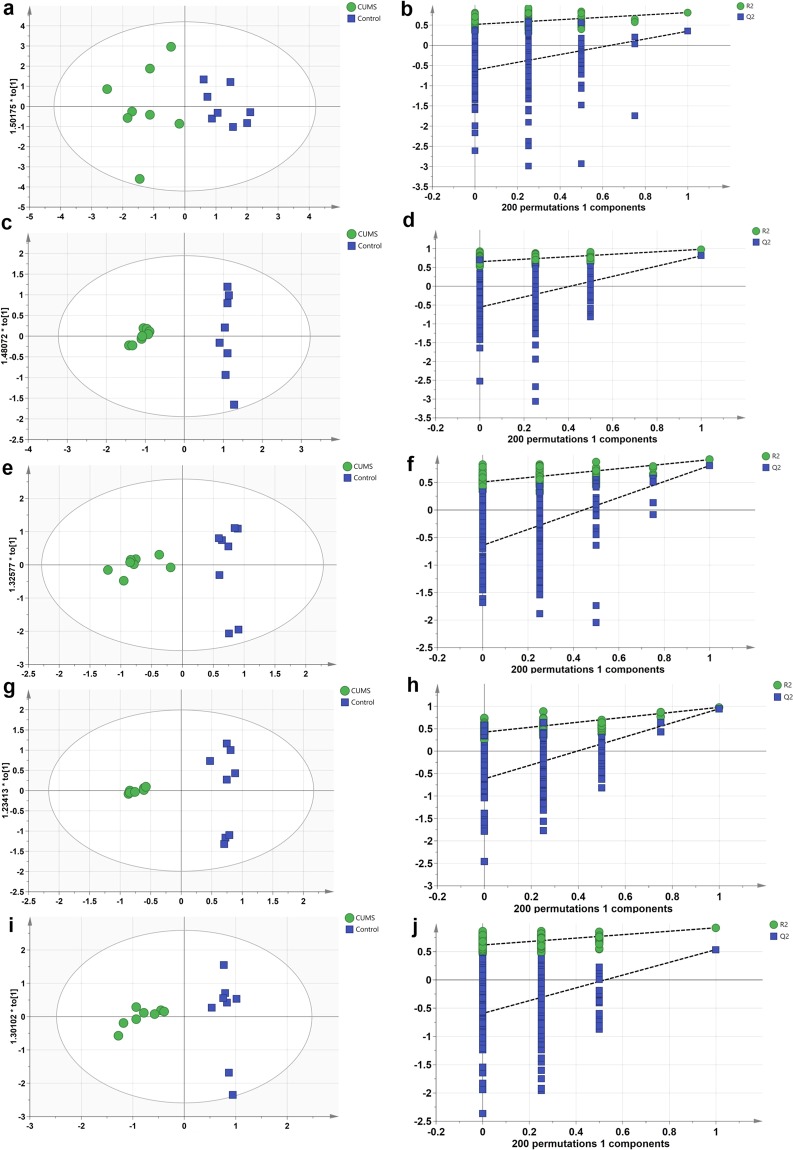
OPLS scores and permutation tests of the OPLS-DA models: serum (**a**,**b**), heart tissue (**c**,**d**), liver tissue (**e**,**f**), brain tissue (**g**,**h**), and kidney tissue (**i**,**j**) samples.

The OPLS-DA analysis and the permutation tests to assess the OPLS-DA models were performed using GC-MS data. There were 10, 10, 9, 4, and 7 differentially expressed metabolites in the serum, heart, liver, brain, and kidney samples, respectively, between the control and CUMS groups (VIP > 1, *p* < 0.05, Table 1). The parameters obtained indicated efficient modeling that clearly separated the CUMS and control groups (serum: R^2^X = 0.761, R^2^Y = 0.812, Q^2^ = 0.35; heart tissue: R^2^X = 0.761, R^2^Y = 0.984, Q^2^ = 0.81; liver tissue: R^2^X = 0.822, R^2^Y = 0.915, Q^2^ = 0.807; brain tissue: R^2^X = 0.802, R^2^Y = 0.977, Q^2^ = 0.945; and kidney tissue: R^2^X = 0.713, R^2^Y = 0.922, Q^2^ = 0.536). Values of these parameters approaching 1.0 indicate a stable model with predictive reliability. The statistical validation using permutation tests to assess the significant OPLS-DA models revealed no over-fitting, as the blue regression line of the Q^2^-points intersects the vertical axis (on the left) all below zero, as shown in Fig. 3.

**Table 1:** List of assigned statistically significant metabolites of the serum, heart, liver, brain and kidney between the CUMS group and the control group.

Metabolites	HMDB	VIP	p-Value	Fold Change
**Serum**				
Oleamide	HMDB0002117	1.75	2.49E-04	5.57E-01
Linoleic acid	HMDB0000673	1.63	1.04E-03	6.84E-01
Urea	HMDB0000294	1.46	4.96E-03	1.97E+00
L-Glutamic acid	HMDB0000148	1.44	6.16E-03	4.69E+00
Palmitic acid	HMDB0000220	1.39	8.61E-03	7.17E-01
D-Lyxose	HMDB0003402	1.38	9.49E-03	3.93E-01
L-Methionine	HMDB0000696	1.33	1.29E-02	4.02E+00
L-Alanine	HMDB0000161	1.33	1.36E-02	5.25E+00
Myristic acid	HMDB0000806	1.31	1.46E-02	7.35E-01
L-Phenylalanine	HMDB0000159	1.26	2.05E-02	2.40E+00
**Heart**				
Phenol	HMDB0000228	1.59	4.75E-07	5.90E+00
L-Valine	HMDB0000883	1.56	1.68E-06	6.58E+00
Urea	HMDB0000294	1.44	6.13E-05	2.21E+00
Glycine	HMDB0000123	1.29	9.72E-04	2.10E-01
L-Alanine	HMDB0000161	1.27	1.14E-03	5.13E+00
L-Threonine	HMDB0000167	1.22	2.28E-03	4.41E+00
D-Lactic acid	HMDB0001311	1.20	3.04E-03	1.93E+00
Stearic acid	HMDB0000827	1.12	6.90E-03	3.98E-01
Propionic acid	HMDB0000237	1.09	8.51E-03	1.75E-01
Uracil	HMDB0000300	1.06	1.12E-02	3.40E-01
**Liver**				
Oxalic acid	HMDB0002329	1.87	2.31E-04	3.18E+00
Uracil	HMDB0000300	1.80	5.40E-04	2.72E-01
Stearic acid	HMDB0000827	1.79	5.74E-04	3.88E-01
Palmitoleic acid	HMDB0003229	1.60	3.69E-03	3.51E-01
Palmitic acid	HMDB0000220	1.44	1.16E-02	5.24E-01
L-Threonine	HMDB0000167	1.35	1.98E-02	2.48E+00
D-Glucose	HMDB0000122	1.31	2.46E-02	1.84E+00
D-Lactic acid	HMDB0001311	1.29	2.67E-02	1.64E+00
Linoleic acid	HMDB0000673	1.23	3.66E-02	4.79E-01
**Brain**				
Cholesterol	HMDB0000067	1.38	1.85E-06	1.50E-01
D-Lactic acid	HMDB0001311	1.28	5.91E-05	5.49E+00
Carbamic acid	HMDB0003551	1.27	6.67E-05	7.97E+00
Stearic acid	HMDB0000827	1.18	5.22E-04	1.11E-01
**Kidney**				
L-Serine	HMDB0000187	1.57	1.18E-04	2.74E-01
D-Glucose	HMDB0000122	1.39	1.47E-03	3.32E+00
L-Isoleucine	HMDB0000172	1.38	1.63E-03	2.74E-01
L-Norleucine	HMDB0001645	1.37	1.97E-03	1.34E-01
Uracil	HMDB0000300	1.28	4.64E-03	1.30E-01
3-Hydroxybutyric acid	HMDB0000357	1.17	1.25E-02	2.73E+00
D-Lactic acid	HMDB0001311	1.14	1.55E-02	1.90E+00

Abbreviations: HMDB: the Human Metabolome Database, Fold change: CUMS/Control, VIP: variable influence on projection.

**Figure 4:**
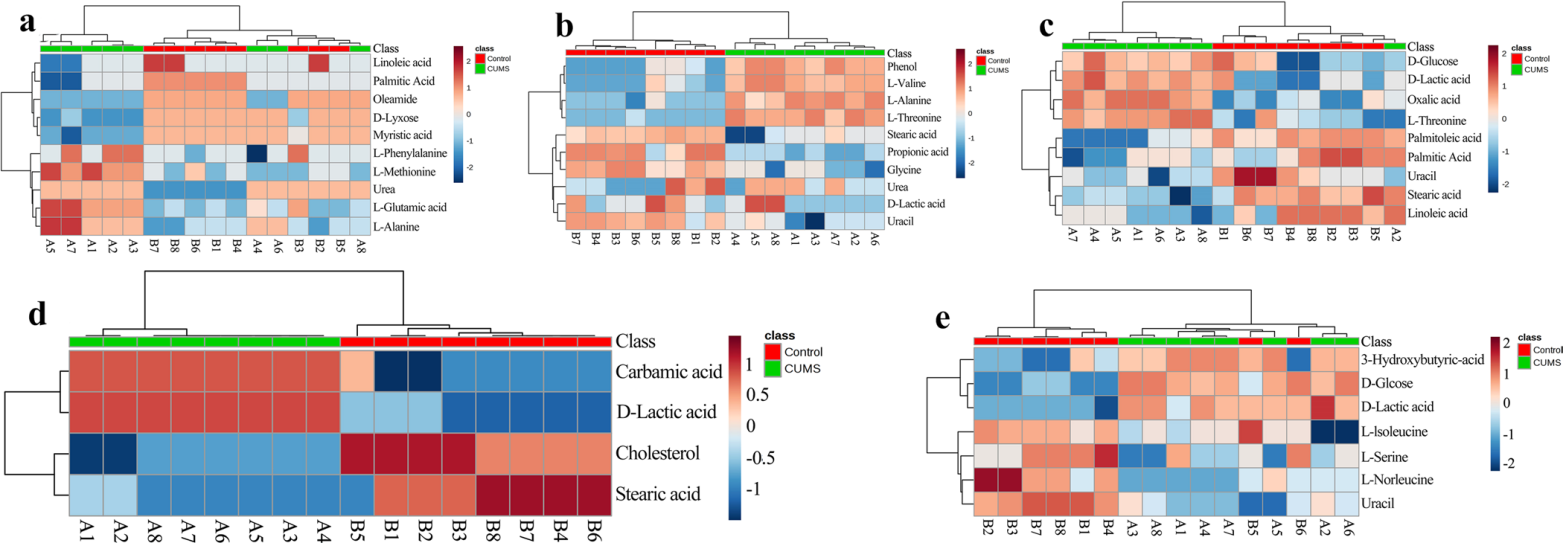
Heatmap of differentially expressed metabolites in the serum (**a**), heart tissue (**b**), liver tissue (**c**), brain tissue (**d**), and kidney tissue (**e**) samples in CUMS rats compared to the controls. The color of each section is proportional to the significance of the change in metabolites (red, up-regulated; blue, down-regulated). Rows correspond to the samples, and columns correspond to the metabolites.

To further understand the metabolic differences between the CUMS and control groups, the data on the identified metabolites were analyzed using MetaboAnalyst 4.0. As shown in Fig. 4, though sample clusters overlapped slightly, most samples were clearly grouped into two differentiated clusters, in agreement with the OPLS analysis.

### Analyses of metabolic pathways

**Figure 5:**
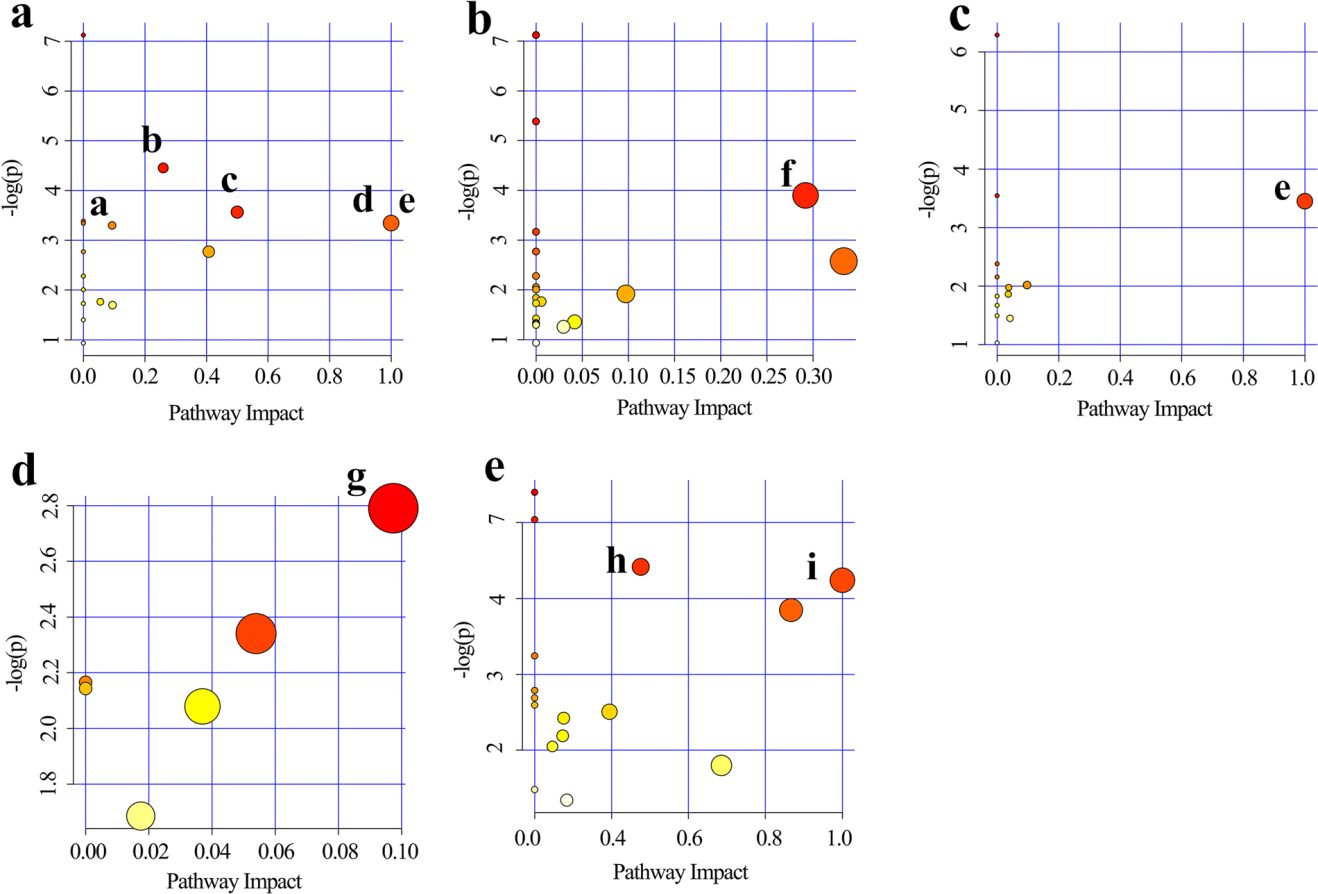
Summary of pathway analysis using MetaboAnalyst 4.0. Serum (**a**): (**a**) alanine, aspartate and glutamate metabolism, (**b**) phenylalanine, tyrosine, and tryptophan biosynthesis, (**c**) D-glutamine and D-glutamate metabolism, (**d**) arginine and proline metabolism, and (**e**) linoleic acid metabolism. Heart tissue (**b**): (**f**) glycine, serine, and threonine metabolism. Liver tissue (**c**): (**e**) linoleic acid metabolism. Brain tissue (**d**): (**g**) pyruvate metabolism. Kidney tissue (**e**): (**h**) aminoacyl-tRNA biosynthesis and (**i**) methane metabolism.

We identified several significant pathways (Raw *p* < 0.5, Impact > 0) (Table 2). The following pathways had the greatest significance in serum: (a) alanine, aspartate and glutamate metabolism, (b) phenylalanine, tyrosine and tryptophan biosynthesis, (c) D-glutamine and D-glutamate metabolism, (d) arginineand proline metabolism, and (e) linoleic acid metabolism; heart tissue: (f) glycine, serine and threonine metabolism; liver tissue: (g) linoleic acid metabolism; kidney tissue: (h) aminoacyl-tRNA biosynthesis and (i) methane metabolism. In addition, pyruvate metabolism was found to have a high impact value in the brain tissue but the result was not significant. The detailed results of the pathway analyses are shown in Table 2, with a summary shown in Fig. 5.

**Table 2:** Results from pathway analysis by MetaboAnalyst 4.0.

Pathway name	Total	Expected	Hits	Raw p	Holm adjust	FDR	Impact
**Serum**							
Alanine, aspartate and glutamate metabolism	24	1.71E-01	2	1.16E-02	0.93018	3.74E-01	2.59E-01
Phenylalanine, tyrosine and tryptophan biosynthesis	4	2.85E-02	1	2.83E-02	1	3.74E-01	5.00E-01
D-Glutamine and D-glutamate metabolism	5	3.57E-02	1	3.52E-02	1	3.74E-01	1.00E+00
Linoleic acid metabolism*	5	3.57E-02	1	3.52E-02	1	3.74E-01	1.00E+00
Arginine and proline metabolism	44	3.14E-01	2	3.69E-02	1	3.74E-01	9.35E-02
**Heart**							
Glycine, serine and threonine metabolism	32	2.28E-01	2	2.03E-02	1	5.47E-01	2.92E-01
**Liver**							
Linoleic acid metabolism*	5	3.21E-02	1	3.17E-02	1	8.57E-01	1.00E+00
**Brain**							
Pyruvate metabolism	22	6.28E-02	1	6.14E-02	1	1.00E+00	9.74E-02
**Kidney**							
Aminoacyl-tRNA biosynthesis	67	3.35E-01	2	4.04E-02	1	8.07E-01	1.38E-01
Methane metabolism	9	4.49E-02	1	4.42E-02	1	8.07E-01	4.00E-01

Abbreviations: FDR: false discovery rate.

## Discussion

Allostatic overload can be caused by chronic stress, and the consequences of allostatic load include increased risk of cardiovascular disease, abnormal hepatic metabolism, atrophy of neurons in the whole brain, and poor kidney outcomes^12–14^. The underlying pathologic mechanisms of diseases associated with allostatic overload may be involved in oxidative stress and systemic inflammation, disturbances in the autonomic nervous system and circadian rhythm, as well as metabolite abnormalities in the main stress-targeted tissues^2,5^.

Depression is a multifactorial disorder, closely associated with allostatic overload^15^. Thus, to provide an overview from the perspective of systematic metabonomics, a non-targeted GC-MS-based metabonomics approach coupled with univariate and multivariate analyses was employed to identify metabolic biomarkers in the main stress-targeted tissues associated with depression. To our knowledge, our study is the first systematic metabonomics study on the whole body to explore the metabolic mechanisms involved in depression, which may assist researchers in understanding the pathogenesis of depression and developing new therapeutic strategies.

**Figure 6:**
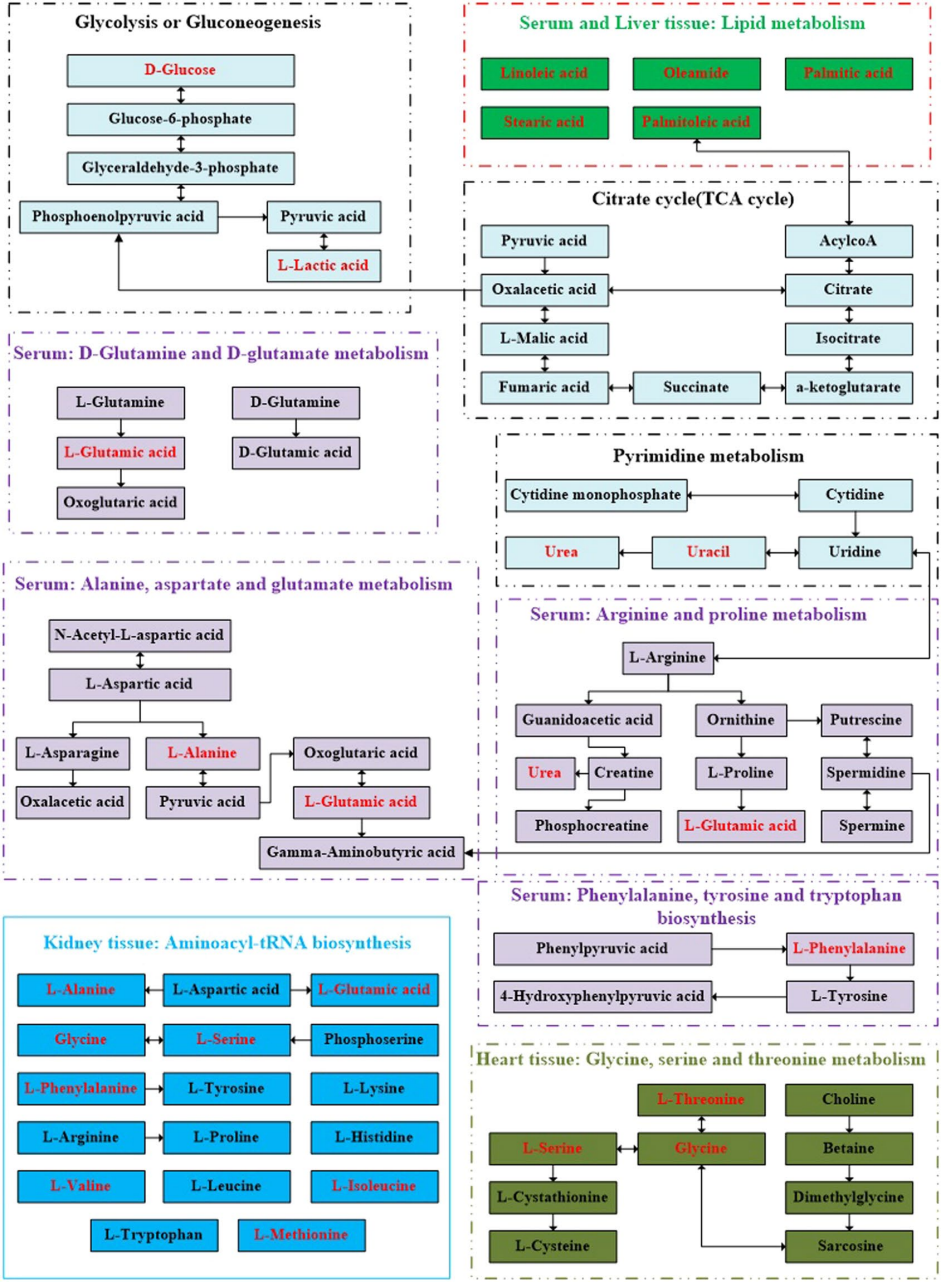
Schematic diagram of the proposed metabolic pathways in main stress-targeted tissues (serum, heart, liver, brain and kidney) of CUMS rats compared to the controls (as shown in different colors). Metabolites marked in red represent the significant biomarkers found in stress-targeted tissues.

A total of 10, 10, 9, 4, and 7 differentially expressed metabolites were identified in the serum, heart, liver, brain, and kidney tissues, respectively, between the control and CUMS groups. As seen in Fig. 5, these metabolite biomarkers were involved in nine significant pathways, which were mainly related to amino acid, lipid, energy, and nucleotide metabolism. As shown in Fig. 6, these biomarkers are closely associated with each other.

Amino acids and their metabolites are basic substrates and regulators in many metabolic pathways and have gained increased attention from more and more researchers^16–18^. Altered amino acid levels have been identified as markers of risk for schizophrenia, Alzheimer’s disease, and type 2 diabetes^19,20^. Accordingly, in our study, we found that L-alanine, L-glutamic acid, glycine, L-methionine, L-phenylalanine, L-valine, L-isoleucine, and L-norleucine were significantly changed in the CUMS group compared to the control group (as measured by GC-MS). These metabolites were involved in the following pathways (a) alanine, aspartate, and glutamate metabolism, (b) phenylalanine, tyrosine, and tryptophan biosynthesis, (c) D-glutamine and D-glutamate metabolism, (d) arginine and proline metabolism, and (f) glycine, serine, and threonine metabolism and the most significant changes may owe to the abnormal metabolism of amino acids. L-alanine was increased in both serum and heart tissue of CUMS rats, and it is thought to be one of the links between depression and cardiovascular disease. L-alanine is directly involved in gluconeogenesis and the alanine–glucosecycle and regulates glucose metabolism. Like gamma-aminobutyric acid, taurine, and glycine, as an inhibitory neurotransmitter, L-alanine is involved in lymphocyte reproduction and immunity, but the association between L-alanine and depression still requires more research. L-glutamic acid, also referred to as glutamate, obtained from the hydrolysis of glutamine, is a key molecule in cellular metabolism. Glutamate and glutamine are precursors of the major natural antioxidant glutathione (GSH), playing important roles in maintaining the cellular redox equilibrium^21^. The serum glutamate was elevated in the serum of CUMS rats in our study and the results was in inconsistent with a previous study^22^, additionally, glutamate was increased in hippocampus but decreased in thalamus and the remaining brain regions in a earlier study also using GC-MS to evaluate four brain regions of chronic unpredictable mild stress-model rats^23^. Overall, the perturbations of glutamate may be due to the disturbances of the glutamine–glutamate cycle, which may partly underlie the pathogenesis of depression. It is still noteworthy that the discrepancy of glutamate should be deserved attention. L-threonine and glycine are involved in glycine, serine, and threonine metabolism. Glycine is a semi-essential amino acid and a basic nutrient. As a component of the endogenous antioxidant GSH, glycine is involved in oxygen stress and the cell membrane injury processes related to depression. In our study, the elevated heart and liver L-threonine and reduced heart glycine were related to the abnormality of glycine, serine, and threonine metabolism. Phenylalanine is an essential amino acid and the precursor of catecholamines, which are neurotransmitters and adrenaline-like substances that play crucial roles in depression. Valine, leucine, and isoleucine are branched-chain amino acids (BCAAs) involved in stress, energy generation, and muscle metabolism. Abnormalities in valine, leucine, and isoleucine levels have been documented in first-episode psychosis. Our study also suggested that abnormal changes in valine, leucine, and isoleucine levels are associated with depression, which was in agreement with a previous study^23^.

All in all, the altered amino acid levels in the serum, heart, liver, brain, and kidney tissues of the CUMS rats may assist researchers in understanding the pathogenesis of depression. The precise mechanisms by which amino acid levels are associated with the genesis and development of depression should be investigated further.

In our study, the levels of linoleic acid, palmitic acid, stearic acid, oleamide, and palmitoleic acid were significantly changed in the CUMS group compared to the control rats. Previous research has shown that alterations in the brainn-6/n-3 polyunsaturated fatty acid (PUFA) ratio and monounsaturated fatty acid (MUFA)/saturated fatty acid (SFA) content (e.g., docosahexaenoic acid, C22:6n-3; oleicacid, C18:1; palmiticacid, C16:0; and stearic acid, C18:0) occur in patients with depressive disorder, which may underlie the pathologic onset and progression^24,25^. In our study, linoleic acid metabolism disturbance was found in both serum and liver tissues. Linoleic acid, a precursor to arachidonic acid (AA), was associated with inflammation, type 2 diabetes and cardiovascular disease^26,27^. It has been well confirmed that inflammation and oxidative stress play a key role in the mechanism of depression. Additionally, nuclear erythroid-related factor 2 (Nrf2) was involved in metabolic homeostasis and inflammation and has become a promising target for the prevention or treatment of depressive disorders. As earlier researches showed that fish oil and conjugated linoleic acid can induce Nrf2 upregulation, their protective ability has been evaluated in a rat model of depression^28,29^. The discovery that the levels of linoleic acid, palmitic acid, stearic acid, oleamide, and palmitoleic acid were significantly altered in the CUMS group compared to the control rats may help to understand the molecular mechanisms associated with inflammation and oxidative stress that underlie depression.

To our knowledge, a disturbance in methane metabolism in the kidney of rats with CUMS-induced depression was discovered for the first time. In the global carbon cycle, methane is principally metabolized by methanotrophs and methanogens. Methanogens can obtain energy for growth by converting a limited number of substrates to methane under anaerobic conditions^30^. In our study, L-serine was involved in methane metabolism, which underscores the importance of L-serine in cell proliferation and growth. Thus, the perturbations of normal methane metabolism in the kidney may adversely influence central nervous system, leading to depression. However, there is no adequate evidence on the effects of methane metabolism in depression, and further studies are needed.

In our study, pyruvate metabolism in the brains of the CUMS rats may be disturbed; although the result was not significant, it is still noteworthy due to its high impact value. Pyruvate is the end-product of glycolysis, a major substrate for oxidative metabolism, and abranching point for glucose, lactate, fatty acid, and amino acid synthesis^31^. The brain is an incredibly complex and highly metabolic organ that is almost completely reliant upon glucose and pyruvate metabolism to generate cellular energy^32^. The brain, which has a high demand for ATP, is most affected out of all the tissues, due to its predominant reliance on carbohydrate metabolism for ATP generation. Aberrant pyruvate metabolism plays an especially prominent role in cancer, heart failure, and neurodegeneration^33^. In our study, pyruvate metabolism may have been linked to depression, but the sample size may have been too small to detect a true significant difference in pyruvate metabolism. Thus, understanding and exploiting pyruvate metabolism may yield novel treatments that enhance human health.

In conclusion, we used a GC–MS platform to characterize the metabolic profiles of the main stress-targeted tissues (serum, heart, liver, brain, and kidney) to comprehensively understand the whole-body response to CUMS-induced depression in rats. The findings highlight classes of metabolites and biochemical pathways that are altered in the whole body in CUMS rats compared to the control rats, helping to understand the pathophysiological mechanisms underlying CUMS-induced depression. However, verification and validation studies with larger independent samples are necessary to demonstrate the utility of these metabolites as potential disease markers, additionally, as we all know, chronic stress involves the whole body, disorder of urogenital system, alimentary system, endocrine system and other systems were also influenced by chronic stress and need further study.

## Methods

### Animals and ethics statement

Eight-week-old male Sprague–Dawley rats (180–240 g) were randomly divided to one of the two groups (CUMS or control), with eight rats each. The study protocol was approved by the Medical Ethics Committee of the Jining First People’s Hospital, Jining Medical University (No. 20170016). All animal procedures were conducted in accordance with the National Institutes of Health guide for the care and use of Laboratory animals.

### Materials and instruments

Heptadecanoic acid (purity: ≥98%; lot: SLBX4162), which was used as an internal standard (IS), and N, O-bis(trimethylsilyl)trifluoroacetamide with 1% trimethylchlorosilane (BSTFA + 1% TMCS; v/v; lot: BCBZ4865) were purchased from Sigma–Aldrich (Saint Louis, MO, USA). Chromatographic-grade methanol was purchased from Thermo Fisher Scientific (Waltham, MA, USA). o-Methyl hydroxylamine hydrochloride (purity: 98.0%; lot: 542171) was purchased from J&K Scientific Ltd., (Beijing, China). Water was purchased from Hangzhou Wahaha Company (Hangzhou, China). Pyridine (lot: C10486013) was purchased from Shanghai Macklin Biochemical (Shanghai, China).

A high-speed tissue homogenizer (KZ-II) was purchased from Servicebio (Wuhan, China). 7890B GC system equipped with a 7000 C mass spectrometer and the separation was on an HP-5MS fused-silica capillary column (30 m × 0.25 mm × 0.25 μm; Agilent Technologies, USA).

### CUMS procedure

CUMS was performed as in our previous study^11^. Briefly, the CUMS treatment was carried out for 4 weeks according to the following conditions: food deprivation (24 h), water deprivation (24 h), 45° cage tilting (24 h), crowed housing (24 h), restraint in an empty water bottle (Wahaha, China) (4 h), noise (20 min), tail clamping (1 min), forced swimming (10 min) and day-night reversal (12 h/12 h). To ensure the procedure unpredictable, these above protocols were randomly scheduled to make the rats receive one of them daily. Behavioral tests-the sucrose preference test (SPT) and the forced swim test (FST) were performed to evaluate the rats for depressive-like states. Results of the behavioral tests were described as means ± SD and GraphPad version 8.0 software was applied to performed t-test.

#### Sucrose preference test (SPT)

The SPT, a method for evaluating anhedonia, before the SPT test, all the individually rats were habituated to taste a 1% sucrose solution in two bottles on left and right side for 48 h. The two pre-weighed bottles with one containing tap water and another containing a 1% sucrose solution, were placed in each cage to rat after 14 h of water deprivation. In order to avoid spatial bias, the two bottles were randomly placed on the left and right side. 1 h later, two bottles were weighed again, and the weight difference was considered as the rat intake in each bottle. Sucrose preference was used as the percentage of the intake sucrose solution/total liquid consumption.

#### Forced swim test (FST)

After the SPT, the FST was conducted, in brief, all rats were individually placed in a 45 cm height, 15 cm diameter of Plexiglas cylinder with water (23–27 °C, 35 cm deep) to conduct a swim test (15 min). Later, all rats were dried and returned to their each cage. Twenty-four hours later, the rats were forced to swim again for 5 min. The two test sessions were videotaped separately and immobility time was assessed by an experienced observer who was blinded to the experimental design in present study.

### Sample collection

Twenty-four hours after the behavioral tests, the rats were anesthetized with 1% sodium pentobarbital (50 mg/kg). Blood was collected and centrifuged at 5000 rpm for 5 min to obtain the serum. The brains were then quickly resected and the rats were rapidly dissected on an ice surface. The whole brain, heart, liver, and kidney samples were washed with 0.9% physiological saline and then all samples were frozen at −80 °C and stored until needed.

### Sample pretreatment for GC–MS

Serum samples: 350 μL methanol containing 100 μg/mL IS was added to 100 μL serum, vortexed, and centrifuged at 14,000 rpm for10 min at 4 °C. The supernatant was transferred to a 2-mL tube and evaporated to dryness at 37 °C under the gentle flow of nitrogen gas. After the extracts were dried, 80 μL o-methyl hydroxylamine hydrochloride (15 mg/mL in pyridine) was added and mixed gently. The solution was incubated for 1.5 h at 70 °C. Subsequently, 100 μL of BSTFA + 1% TMCS was added to each sample, followed by incubation for 1 h at 70 °C. The solution was then vortexed, centrifuged at 14,000 rpm for 2 min at 4 °C, and filtered through a 0.22-μm filter membrane before GC–MS analysis.

Tissue samples: 50 mg tissue (heart, liver, brain, and kidney) was added to a 2-mL tube homogenizer with 1 mL methanol and 50 μL 1 mg/mL IS, homogenized evenly, and transferred to a 2-mL tube. The mixtures were subsequently centrifuged at 14,000 rpm for10 min at 4 °C. Next, 800 μL supernatant was transferred into a 2-mL tube and evaporated to dryness at 37 °C under the gentle flow of nitrogen gas. Subsequently, 80 μL o-methyl hydroxylamine hydrochloride (15 mg/mL in pyridine) was added to the tube and incubated in a water bath at 70 °C for 1.5 h, followed by adding 100 μL BSTFA + 1% TMCS and incubation for a further 1 h at 70 °C to create a derivatized solution. The solution was then vortexed, centrifuged at 14,000 rpm for 2 min at 4 °C, and filtered through a 0.22-μm filter membrane before GC–MS analysis.

### GC–MS analyses

Quality control (QC) of each serum and tissues samples (heart, liver, brain, and kidney) were defined as a mixture of each tissue from the CUMS and control rats. The retention time (RT) stability was assessed using the IS. GC–MS was conducted using a 7890B GC system equipped with a 7000C mass spectrometer. Separation of serum, heart, liver, brain, and kidney samples was carried out using a HP-5MS fused-silica capillary column, and each 1-μL aliquot of derivatized solution was run in split mode (50:1), with helium as the carrier gas and a front inlet purge flow of 3 mL/min; the gas flow rate was 1 mL/min. The GC temperature program began at 60 °C for 4 min, increased to 300 °C at 8 °C/min, and ended with a final 5-min maintenance at 300 °C. The temperatures associated with the injection, transfer line, and ion source were 280 °C, 250 °C, and 230 °C, respectively. Electron impact ionization (−70 eV) was used, with an acquisition rate of 20 spectra/s in the MS setting. MS detection was conducted by electrospray ionization (ESI) in full-scan mode involving mass/charge (m/z) values of 50–800.

### Multivariate statistical analyses

Metabolites were first explored using GC–MS, involving deconvolution, alignment, and data reduction to produce a list of m/z and RT pairs, with the corresponding intensities for all detected peaks from each data file in the dataset. The resulting table was exported into Excel™(Microsoft, Redmond, WA, USA) and normalized. The sample names (observations) and normalized peak area percentages were imported into SIMCA-P 14.0 (Umetrics, Umea, Sweden) for statistical analyses. In the orthogonal partial least squares discriminant analysis (OPLS-DA) models^34^, variable importance in projection (VIP) values >1.0 were considered potentially relevant for group discrimination, and two-tailed Student’s t-test differences of p < 0.05 were considered significant. “Fold-change” was defined as the average mass response (area) ratio between the CUMS and control groups. MetaboAnalyst 4.0 (http://www.metaboanalyst.ca) and the Kyoto Encyclopedia of Genes and Genomes (KEGG; http://www.kegg.jp) were used to assist in the pathway analysis^35^, and Raw p < 0.5, Impact > 0 were defined as significant, which may help to the biochemical interpretation of the metabolites.
